# Case Report: Multiple organ dysfunction syndrome in a preterm infant secondary to respiratory syncytial virus and bacterial co-infection

**DOI:** 10.3389/fped.2026.1825002

**Published:** 2026-05-18

**Authors:** Xue Han, Li Zhang, Rui Zhang, Wenli Liu

**Affiliations:** 1 Department of Neonatology, West China Second University Hospital, Sichuan University, Chengdu, China; 2 Key Laboratory of Birth Defects and Related Diseases of Women and Children, Sichuan University, Ministry of Education, Chengdu, China; 3 Department of Pediatric Respiratory and Immunology, West China Second University Hospital, Sichuan University, Chengdu, China

**Keywords:** case report, mechanical ventilation, metagenomic next-generation sequencing, preterm infant, respiratory syncytial virus

## Abstract

This article reports a case of a 1-month 11-day-old preterm infant, born at 36 + 6 weeks gestation, who presented to an outside hospital emergency department with a persistent cough that had not improved over four days. During this period, the infant progressively developed respiratory distress and lethargy. The infant subsequently developed cardiopulmonary arrest, underwent cardiopulmonary resuscitation, and was transferred to our hospital under endotracheal intubation with positive pressure ventilation. Respiratory pathogen polymerase chain reaction testing of a throat swab was positive for respiratory syncytial virus (RSV), while sputum and bronchoalveolar lavage fluid culture and blood metagenomic next-generation sequencing (mNGS) detected *Haemophilus influenzae* and *S. pneumoniae*. After 22 days of hospitalization and treatment including invasive mechanical ventilation, antibiotic adjustment, intravenous immunoglobulin (IVIG), and dexamethasone, the infant was discharged without further complications. Metagenomic next-generation sequencing provides rapid diagnostic evidence for mixed infections, while integrated interventions, including IVIG, short-course corticosteroids, and nutritional support, effectively modulate immune responses.

## 1 Introduction

Respiratory syncytial virus (RSV) is the leading cause of severe lower respiratory tract infections in neonates, with approximately 20% of infected infants requiring hospitalization. RSV infection can disrupt the airway mucosal barrier, increasing the risk of secondary bacterial co-infections. We report a preterm infant with RSV infection, in whom *Haemophilus influenzae* and *S. pneumoniae* were identified using both conventional diagnostics from sputum and bronchoalveolar lavage fluid (BALF) samples and mNGS from blood sample. The infant developed respiratory failure and cardiopulmonary arrest but showed clinical improvement following multimodal supportive therapy including mechanical ventilation, antibiotic adjustment, IVIG, and corticosteroids.

## 2 Case presentation

A 1-month 11-day-old male infant was admitted with a 4-day history of cough that had worsened in the preceding 24 h, accompanied by tachypnea, dyspnea, and lethargy. The cough progressively worsened, and one day before admission, the infant without fever developed respiratory distress, poor feeding, and decreased activity. While being evaluated at a local emergency department, he experienced frequent apnea; auscultation revealed absence of cardiac activity, prompting immediate resuscitation including endotracheal intubation, cardiopulmonary resuscitation, and intravenous epinephrine. After return of spontaneous circulation and resumption of spontaneous breathing, he was transferred to our hospital under endotracheal intubation with positive pressure ventilation. The infection occurred during the winter season, which is a known period of increased viral transmission. Additionally, the patient had a relevant exposure history, as a family member had recently experienced symptoms of the common cold, suggesting a possible source of contagion, although the specific pathogen was not identified. The infant was delivered via cesarean section at 36 ^+^ ^6^ weeks gestation to a gravida 2, para 3 mother, with a birth weight of 2,935 g and Apgar scores of 10 at both 1 and 5 min.

Physical Examination: Temperature 38 °C; heart rate 160 beats/min; respiratory rate 80 breaths/min; oxygen saturation 92%; blood pressure 69/40 mmHg; weight 3,000 g. Auscultation revealed coarse breath sounds bilaterally with fine crackles in both lungs. The liver edge was palpable 2.5 cm below the right costal margin. Cardiac and abdominal examinations were otherwise normal.

Auxiliary Examinations: C-reactive protein 35.3 mg/dL. Biochemistry: alanine aminotransferase 932 U/L; aspartate aminotransferase 506 U/L; albumin 26.5 g/L. B-type natriuretic peptide 1,729.41 pg/mL. Coagulation studies were essentially normal. Arterial blood gas analysis: pH 7.158; PaCO₂ 61.6 mmHg; PaO₂ 75.7 mmHg; base excess −7.4 mmol/L; lactate 0.6 mmol/L. Respiratory pathogen nucleic acid testing was positive for RSV. Sputum culture, BALF culture, and plasma mNGS detected Haemophilus influenzae and S. pneumoniae. Blood culture and blood RNA mNGS were negative. Cerebrospinal fluid analysis was unremarkable. Chest computed tomography (Day 11) revealed patchy opacities and areas of consolidation involving multiple lobes bilaterally. Bronchoscopy showed pulmonary infection with endobronchitis and type III mucus characteristics (1).

## 3 Diagnostic assessment

Based on the history, clinical manifestations, laboratory pathogen identification, and imaging findings, the diagnosis was established as "Severe pneumonia (with co-infection by RSV, *Haemophilus influenzae* and *S. pneumoniae*), multiple organ dysfunction syndrome involving respiratory failure, hepatic injury, internal environment disorder, and hypoalbuminemia."

**Figure 1:**
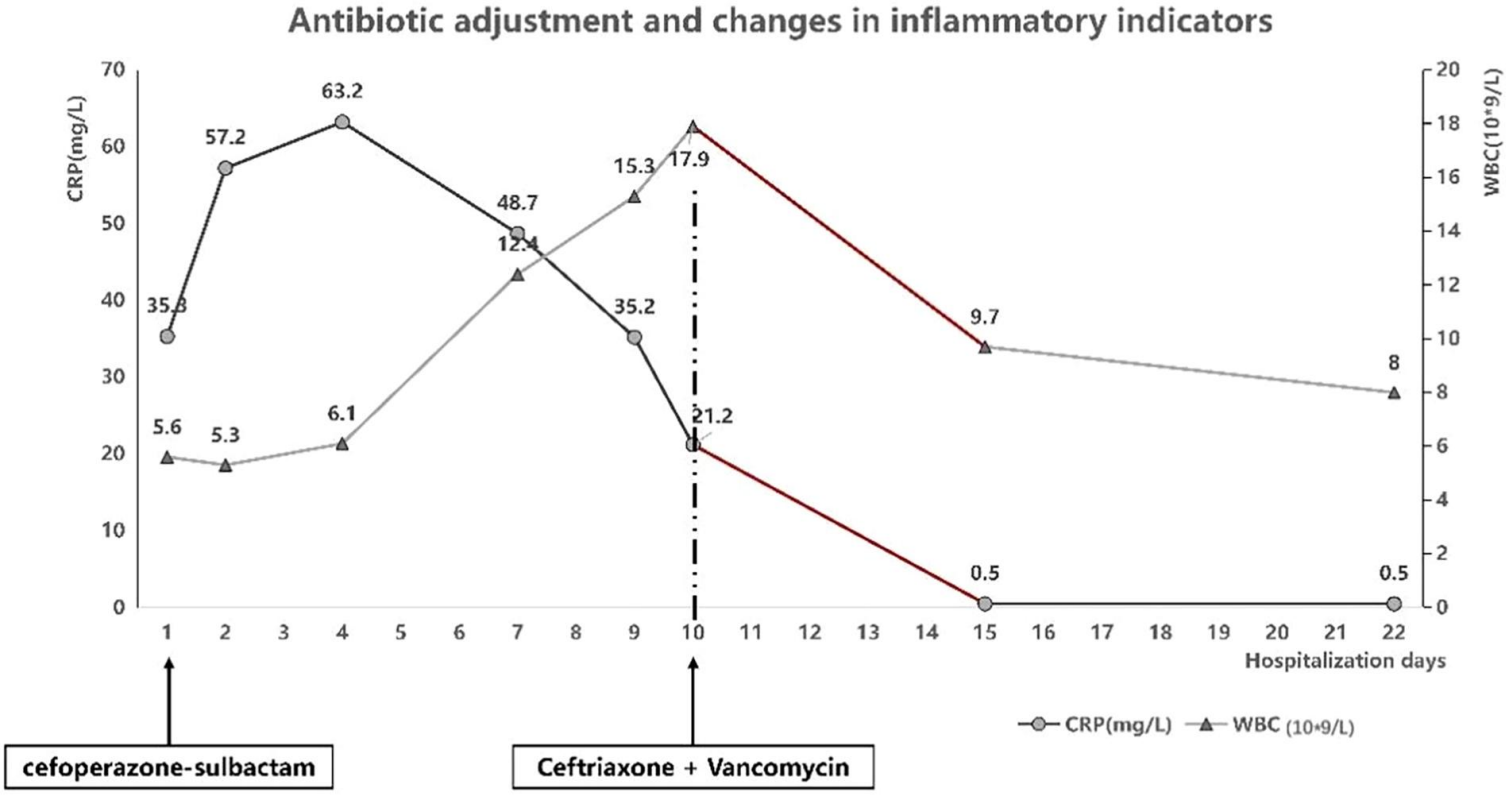
Antibiotic adjustment and changes in inflammatory indicators.

**Figure 2:**
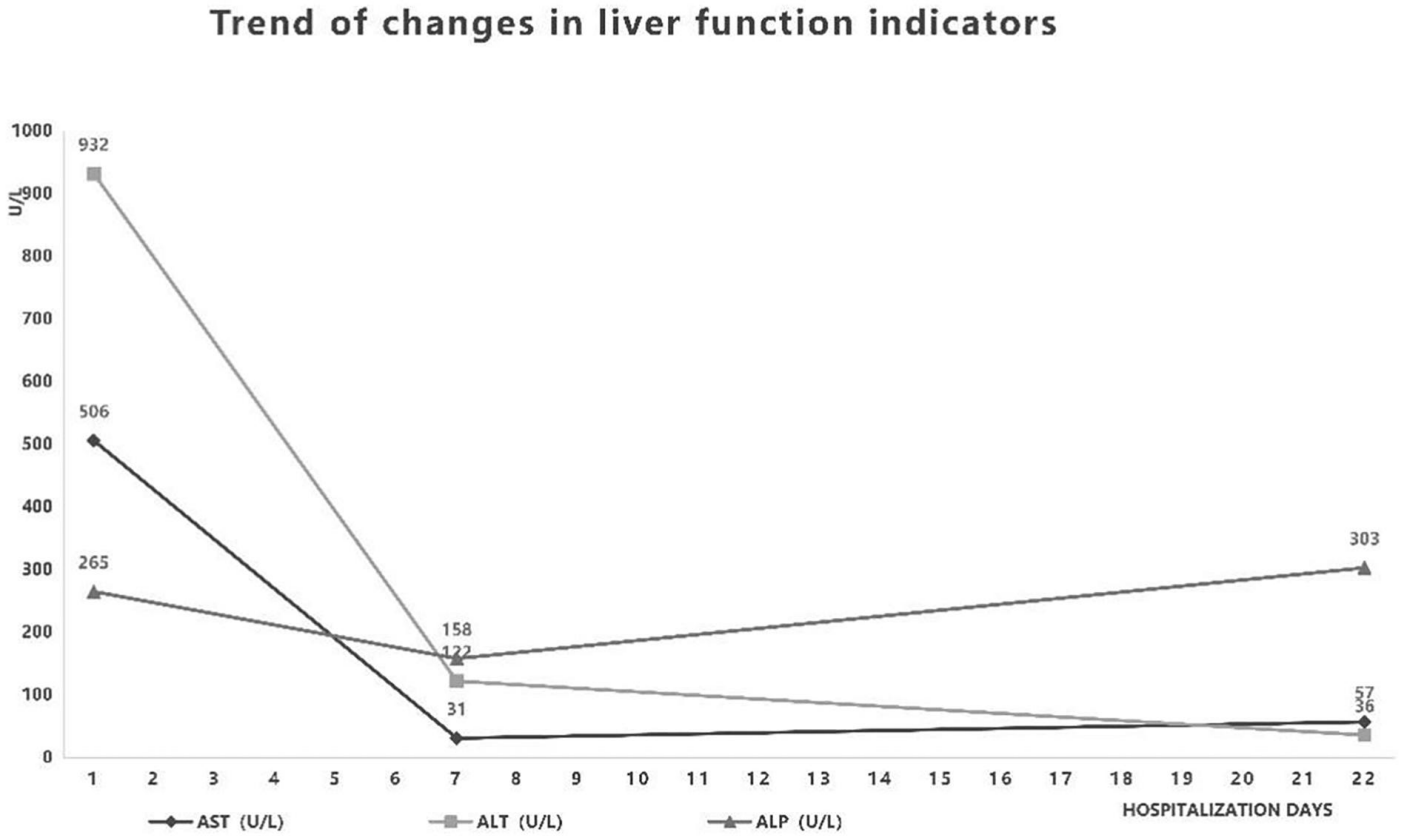
Trend of changes in liver function indicators.

Post-admission management included: 1. Multi-organ support: ①Respiratory support: Due to tachypnea with subcostal recession and high oxygen requirements, invasive mechanical ventilation was initiated: synchronized intermittent mandatory ventilation (SIMV) + pressure support ventilation (PSV) + volume guarantee (VG); VG: 4 mL/kg, PEEP: 6 cmH₂O, RR: 30 breaths/min, Ps: 20 cmH₂O, supplemented with nebulized acetylcysteine, mechanical chest physiotherapy, and prone positioning. Ventilatory support was sequentially transitioned to non-invasive ventilation and ultimately weaned as oxygenation improved and respiratory parameters stabilized (Table 1). ② Concurrent hepatoprotective therapy, along with correction of electrolyte/metabolic imbalances and hypoalbuminemia, contributed to the gradual normalization of liver function. The improvement of liver function was also directly related to the improvement of circulation and oxygenation. 2. After admission, the patient experienced temperature fluctuations and underwent anti-infection and immune regulation: ① Antiviral: nebulized interferon. ② Antibiotic administration: empirical therapy commenced with cefoperazone-sulbactam (Sulperazone), later escalated to ceftriaxone plus vancomycin guided by microbiological results, for a 14-day course, which correlated with subsequent resolution of fever and declining inflammatory markers (Figure 1). ③ Immunomodulation/anti-inflammatory: IVIG 2 g/kg for toxin neutralization and immunomodulation; dexamethasone administered per the DART protocol for anti-inflammatory effect. ④ Parenteral and enteral nutrition: transitioned from parenteral nutrition to full enteral feeding. Following 22 days of treatment, liver function tests (Figure 2) and BNP normalized, oxygenation improved, and the patient was successfully extubated after 14 days of invasive ventilation. The infant was weaned off from nasal cannula and discharged after 22 days with a weight of 3,990 g. Follow-up cranial MRI detected no abnormalities. At present, the child is more than 1 year old, and there is no difference in growth and development with children of the same age.

**Table 1:** Serial respiratory support and blood gas analyses.

Days	Mode	FiO2 (%)	VG (mL)	PIP (cmH2O)	PEEP (cmH2O)	RR (b/min)	pH	PaCO2 (mmHg)	PaO2 (mmHg)	Lac (mmol/L)	SaO2 (%)
1	SIMV + PSV + VG	80	12	/	6	30	7.158	61.6	75.7	0.6	88.7
		60	14	/	6.5	30	7.188	61.8	80.6	0.5	90.8
3	SIMV + PSV + VG	40–60	16	/	6.5	30	7.434	36.9	82.3	1.1	93.8
4	SIMV + PSV + VG	40–60	16	/	7	30	7.433	45.6	53.8	0.7	91.1
9	SIMV + PSV + VG	40–50	18	/	7	30	7.451	49.6	50.0	0.7	90.7
14	SIMV + PSV + VG	35	20	/	5	30	7.382	46	69.2	0.6	95.0
15	NIPPV	40	/	12	6	30	/	/	/	/	/
16	NIPPV	30	/	10	5	30	/	/	/	/	/
17	Nasal cannula	25–30	/	/	/	/	/	/	/	/	/
19	Nasal cannula	21–25	/	/	/	/	/	/	/	/	/
21	Discontinued	/	/	/	/	/	/	/	/	/	/

FiO2, Fraction of Inspired Oxygen; NIPPV, Nasal Intermittent Positive Pressure Ventilation; PaCO2, Partial Pressure of Carbon Dioxide; PaO2, Partial Pressure of Oxygen; lac, Lactate; PEEP, Positive End-Expiratory Pressure; PSV, Pressure Support Ventilation; RR, Respiratory Rate; SaO2, Arterial Oxygen Saturation; SIMV, Synchronized Intermittent Mandatory Ventilation; VG, Volume Guarantee; PIP, Peak inspiratory pressure.

## 4 Discussion

Acute lower respiratory infections are among the leading causes of illness and death worldwide in children under 5 years of age, with RSV being the most common pathogen (2, 3). Due to lung immaturity and impaired immunity, preterm infants and those aged ≤3 months represent a high-risk group for severe RSV infection (4). Increasing evidence shows that the widespread use of RSV vaccines and monoclonal antibodies in newborns or infants is associated with improved clinical outcomes. In many countries, including the United States, there are strategies for providing pregnant women with RSV immunization (passive immunity) to offer protection to newborns before they receive their own immunizations (5, 6). These interventions can provide passive immunity, significantly reducing RSV-related healthcare use, hospitalization rates, and thereby easing the healthcare burden (7–9).

Laboratory testing in this case, including respiratory pathogen nucleic acid testing, sputum and BALF culture, and blood mNGS, confirmed co-infection with RSV, *Haemophilus influenzae*, and *S. pneumoniae*. Several studies and expert consensus recommend that mNGS in BALF or sputum supernatant should be considered for pneumonia patients. In this patient, although the same bacteria were identified by culture in both sputum and BALF samples, the blood culture was negative. Therefore, we performed mNGS on a blood sample to assess whether the patient with severe pneumonia had bloodstream infection with the same pathogens. Interestingly, *Haemophilus influenzae* and *S. pneumoniae* were also detected by blood mNGS. These findings demonstrate that mNGS is more effective than traditional culture methods in detecting and diagnosing bacteremia, especially when the bacterial load is low. Furthermore, mNGS supports a shift from empirical antibiotic use to targeted treatment strategies, helping to prevent antibiotic overuse and enabling the precise identification and management of multiple pathogens in complex infections.

Research indicates that RSV infection is largely confined to ciliated epithelial cells lining the airway, with occasional involvement of non-ciliated cells and rare cases of viremia. This localized tropism explains why blood mNGS was negative for RNA viruses in our case, a finding consistent with typical RSV pathogenesis and providing important clinical distinction from other respiratory viruses (e.g., adenovirus) that often cause systemic dissemination.

Severe RSV infection often involves extensive infiltration of immune cells and widespread destruction of the airway epithelium (10), which is consistent with this patient's predominant respiratory symptoms and the absence of multisystem inflammatory syndrome (MIS). This localized pathology was further supported by two key laboratory findings: normal thrombin-antithrombin complex levels, indicating no systemic coagulopathy, and negative blood mNGS for RNA viruses, confirming the absence of viremia. In contrast, pathogens such as adenovirus or COVID-19 mainly target alveolar epithelial cells. Their interaction with the dense capillary network and host immune-mediated viral clearance mechanisms frequently promotes disseminated infection, which may trigger MIS and induce hypercoagulability (11, 12).

RSV infection increases blood-brain barrier permeability, allowing immune cell infiltration into the central nervous system. This process can lead to neurological complications, including seizures, central apnea, and encephalopathy (13). In this patient, cardiorespiratory arrest at the referring facility was attributed to RSV-associated central apnea combined with secondary apnea caused by airway secretion obstruction. Additionally, laboratory tests showed a disproportionate elevation in ALT, AST, and ALP (Figure 2), with normal direct bilirubin levels. This pattern suggests acute ischemic hepatocellular injury, likely secondary to hypoxemia caused by respiratory failure and cardiorespiratory arrest. With restoration of adequate perfusion and oxygenation, a rapid recovery of hepatic enzyme levels is expected (14).

**Figure 3:**
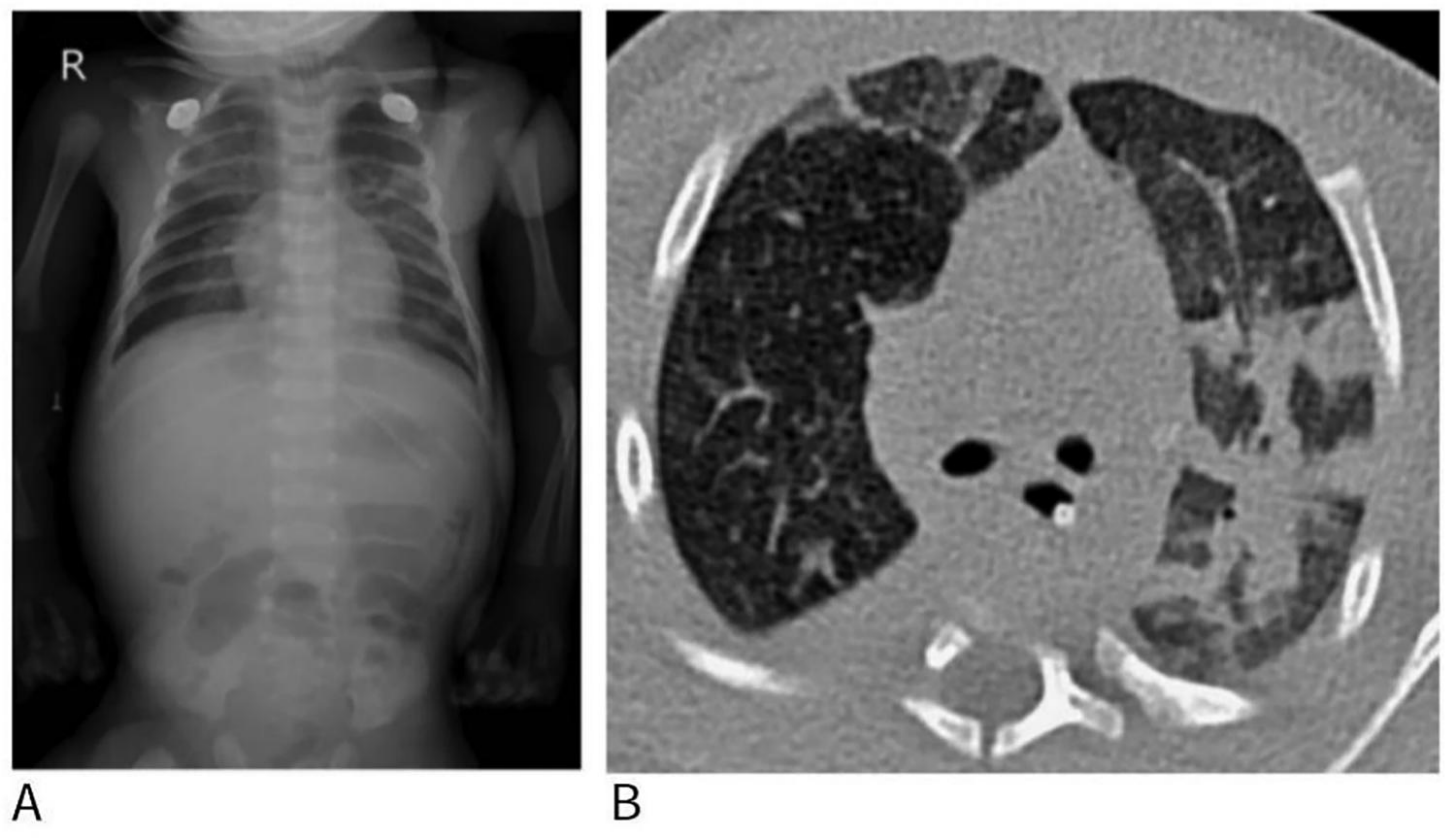
Chest radiograph and CT revealed bilateral multifocal infiltrates. **(A)** Chest radiograph obtained at admission; **(B)** CT scans taken on the 11th day of hospitalization.

In severe RSV pneumonia, inflammatory airway narrowing and mucus plugging increase airway resistance, raising the risk of patient-ventilator asynchrony, incomplete exhalation, elevated intrinsic PEEP, and ventilator-induced lung injury during mechanical ventilation (15). In this case, a combined SIMV + PSV mode preserving spontaneous breathing was used to improve patient-ventilator synchrony and comfort. To address heterogeneous lung pathology (Figure 3) and the coexistence of fast- and slow-responding alveolar compartments, ventilator settings were optimized as follows: a prolonged inspiratory time during SIMV cycles was set to facilitate filling of slow alveoli and promote homogeneous gas distribution, while an extended expiratory time was applied to ensure adequate gas emptying. Patient-triggered PSV breaths were used to accommodate the fast-responding alveoli (16) VG was added to dynamically adjust PIP based on real-time respiratory mechanics, maintaining consistent tidal volumes and reducing ventilator-induced lung injury. Ventilator waveforms were closely monitored to optimize the inspiratory-to-expiratory (I:E) ratio. Recruitment maneuvers with PEEP titration were performed when indicated. Prone positioning was concurrently applied to promote secretion drainage, optimize the ventilation/perfusion ratio, and improve oxygenation (17).

## 5 Conclusion

RSV-bacterial coinfection is an important cause of severe pneumonia in preterm infants. Early pathogen identification, appropriate antibiotic selection, and multimodal supportive therapy are essential for improving outcomes. mNGS provides rapid diagnostic evidence for mixed infections, while integrated interventions, including IVIG, short-course corticosteroids, and nutritional support, effectively modulate immune responses. Strengthened preventive awareness against RSV infection in high-risk populations, especially high-risk preterm infants, is crucial to reduce disease incidence, mitigate severe complications, and improve long-term prognoses.

## Data Availability

The original contributions presented in the study are included in the article/Supplementary Material, further inquiries can be directed to the corresponding author.
